# A Narrative Review on the Effect of Maternal Hypothyroidism on Fetal Development

**DOI:** 10.7759/cureus.34824

**Published:** 2023-02-09

**Authors:** Arundhati Pande, Ashish Anjankar

**Affiliations:** 1 Biochemistry, Jawaharlal Nehru Medical College, Datta Meghe Institute of Medical Sciences, Wardha, IND

**Keywords:** pregnancy, iodine, development, hormones, thyroid

## Abstract

The thyroid is a butterfly-shaped gland located in the human body's neck region. The thyroid produces three hormones that are essential for regulating body temperature, energy production, weight, hair and nail growth, and menstrual cycle maintenance. The production of these hormones is controlled by a feedback mechanism. Various factors cause changes in the stimulation and inhibition of these hormones, which ultimately causes either excessive release or a decrease in the levels of thyroid hormones. These causes can be physiological or pathological. One of the physiological causes is pregnancy. Pregnancy is a very complex process in which many changes occur in the body and its functioning. One of which is changes in the maternal thyroid gland. The inability to adequately adapt to the changes leads to the abnormal functioning of the thyroid gland. During pregnancy, there is a variation in the concentration of thyroid hormones which may cause a decrease in levels or inhibition in the production of thyroid hormones. This condition is called hypothyroidism. Hypothyroidism in pregnant mothers can either be gestational or may be a condition that is present way before her pregnancy. Often, gestational hypothyroidism reverts after delivery during the postpartum period but can also be present as subclinical hypothyroidism. In such cases, they pose a significant threat to development, cause growth hindrance to the infant in the womb, and cause abnormalities in the offspring in the future. Some of the changes occur in the gland because of enhancement in levels of thyroid binding globulin, increased clearance rate of iodine from the body in kidneys, altered effects in human chorionic gonadotropin hormone, and decreased consumption of iodine in meals. Iodine disbalance in maternal hypothyroidism is associated with severe health issues like cretinism and mental retardation. Thyroid hormones are crucial for the infant's neural, cognitive, and intelligence quotient development in the womb. Thus, the disturbances in the maternal hormone levels disturb typical early developmental characteristics. In the world of rapidly advancing scientific research, there are many ways in which this condition can be detected early, diagnosed correctly, and given apt and required attention and treatment for causing the least harm to the fetus and the mother.

## Introduction and background

Pregnancy, a natural physiological process, is a stressful condition for the body. It is accompanied by hormonal and metabolic changes within the body, which may result in numerous pathophysiological processes and can have a tendency to pose severe outcomes if left untreated [1,2]. One organ that undergoes physiological changes during pregnancy is the thyroid gland. These specific changes alter the onset and further progression of pregnancy [3]. In pregnancy, the thyroid gland function is observed by a T4 surge at the 12th week which gradually declines. In serum, thyroid hormone levels fall during the second half of pregnancy. So, diagnosing hypothyroidism during pregnancy is challenging due to variations in thyroid hormone levels in the blood [4,5]. During pregnancy, some factors are responsible for lowering the levels of these hormones in the blood. As a result, moderate or severe deficiency of thyroid hormones can be observed. Depending on the levels of these hormones, the condition can be classified as clinical hypothyroidism, subclinical hypothyroidism, and isolated hypothyroxinaemia [3,6]. Subclinical hypothyroidism (SCH) is a condition where there is an elevation of TSH concentration levels with normal levels of thyroxine (T4) hormone in blood serum. The usual range of TSH during pregnancy is now re-established to 2.5 mlU/L during the first three months of pregnancy and 3.0 mlU/L during the remaining six months [7,8]. Sufficient levels of this hormone are necessary for average growth and development [9,10]. Brain development depends on the maternal iodine supply, which transports T4 into the fetus, thus proving the importance of the proper amount of iodine in the diet [4,5]. Hence, optimal levels of thyroid hormones are necessary for the proliferation and differentiation of cytotrophoblasts. The most common cause of gestational hypothyroidism is a deficiency in iodine levels [11]. It causes several health effects, especially in mothers and infants [9]. Hashimoto’s disease, an autoimmune disorder, is one of the most common reasons for primary hypothyroidism in which the thyroid is attacked by its own body's immune system interfering with the normal functioning of the thyroid hormones [1,12]. Maternal hypothyroidism can lead to a higher risk of fetal hypothyroidism [4,13,14]. It needs careful management as this abnormality can hinder mental development and may cause compressive goiter in infants. In morphological thyroid disorders, the problem is usually differentiated thyroid cancer, as its frequency of growth is higher during pregnancy. Its consequences are aggravated and enhanced by the thyroid-stimulating hormone (TSH), like the effect of the human chorionic gonadotropin (hCG) hormone [4,14].

## Review

Search methodology

**Figure 1:**
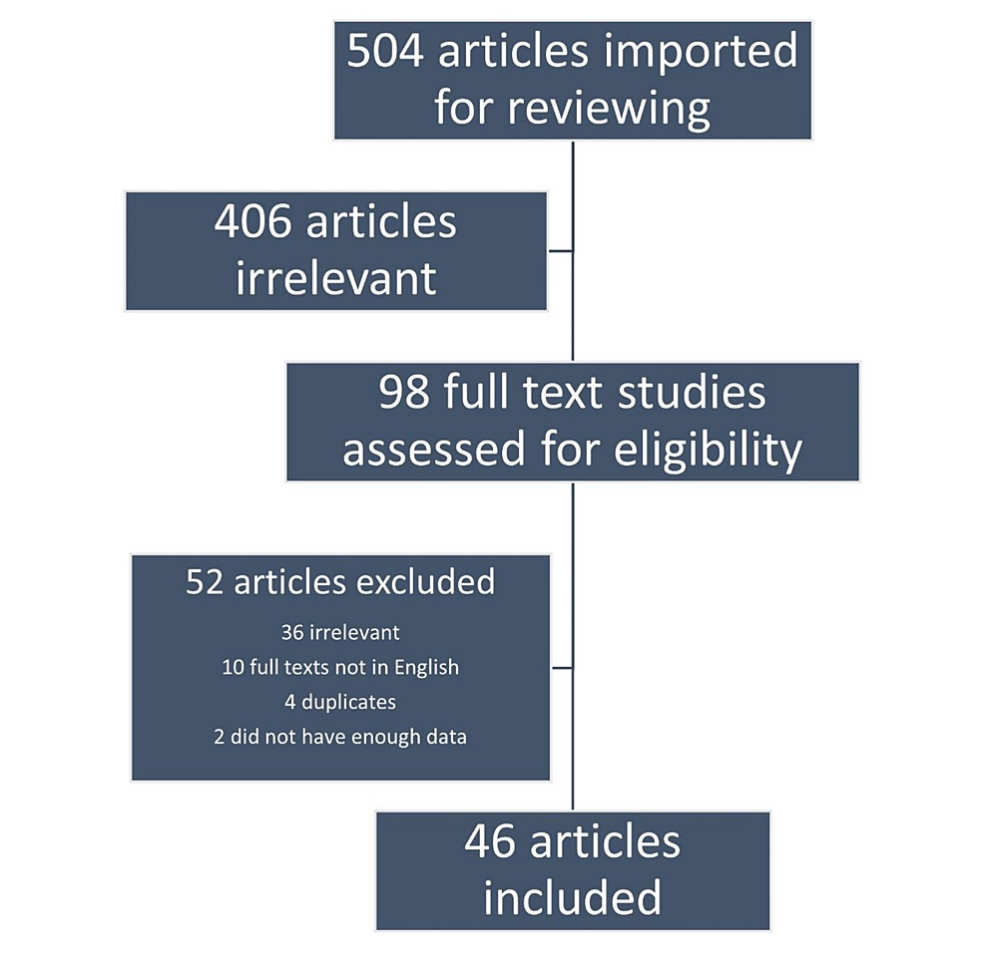
Screening and the number of articles included in the final review.

The strategies used to make this review article include considering research articles published in journals indexed in reputed, reliable, and authentic platforms, processing articles according to different systems, and framing the review like a discussion section of an article where details are explained in straightforward sentences. The Databases searched were PubMed, Google Scholar, and Web of Science. Articles published within 15 years were included for review. Inclusion criteria included females suffering from hypothyroidism during the pregnancy. Exclusion criteria include hypothyroidism with comorbidities such as cardiovascular disease and other secondary pathologies. Key terms used for the search are (maternal hypothyroidism[Title/Abstract] OR hypothyroidism in pregnancy[Title/Abstract] OR hypothyroidism during pregnancy[Title/Abstract] OR subclinical hypothyroidism in pregnancy[Title/Abstract] OR thyroid hormones during pregnancy[Title/Abstract] OR thyroid disorders in pregnancy[Title/Abstract] OR overt hypothyroidism during pregnancy[Title/Abstract] OR maternal thyroid deficiency[Title/Abstract] OR maternal hypothyroidism[MeSH Terms] OR hypothyroidism[MeSH Terms]) AND (fetal development[Title/Abstract] OR fetal neurological development[Title/Abstract] OR foetal development[Title/Abstract] OR foetal neurological development[Title/Abstract] OR fetal development[MeSH Terms] OR foetal development[MeSH Terms]). Screening and the number of articles included in the final review are summarized in Figure 1.

Thyroid hormone in fetal development

**Figure 2:**
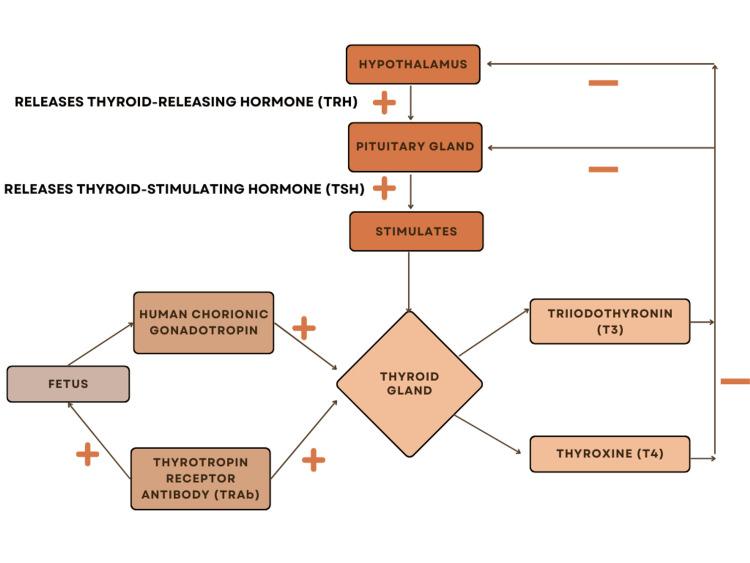
Thyroid hormone regulation in the fetus. Figure Credits: Arundhati Pande (Author)

About 20 years ago, many endocrinologists brought the effects of maternal thyroid hormone deficiency to public attention [15]. In 1999, it was demonstrated that a child's neurodevelopment might be severely affected if hypothyroidism is left untreated in pregnant women [16]. For normal fetal development, an adequate quantity of thyroid hormone is necessary [17]. By 10-12 weeks of gestation, the fetal thyroid gland develops and produces the thyroid hormone. Until the fetus reaches 36 weeks of gestation, thyroid hormone levels in serum do not reach the levels of an adult [18]. As the thyroid hormone can cross the placenta in the first trimester, the fetus depends on its mother for hormone transport [19]. During the early phase of pregnancy, the hCG hormone has a weak stimulatory effect on the thyroid gland [20]. As a result, the maternal serum thyrotropin (TSH) levels decrease, which then subsequently increase. Consequently, maternal serum thyrotropin (TSH) levels fall, followed by a rise in free thyrotropin levels [21,22]. If the mother is a known case of hypothyroidism throughout the pregnancy, then there is a very high risk of low-birth-weight babies being born [23]. TSH receptor-stimulating antibodies and TSH receptor-blocking antibodies are the two types of TSH receptor antibodies. The capacity of maternal TSH receptor antibodies to penetrate the placenta results in impaired fetal thyroid function [24]. Figure 2 depicts thyroid hormone regulation in the thyroid gland of the fetus.

Factors associated with the development of maternal hypothyroidism and its metabolism during pregnancy

The following are major risk factors for developing maternal hypothyroidism: history of thyroid dysfunction, goiter, thyroid antibody positive, age above 30 years, type 1 diabetes mellitus, previous thyroid surgery, autoimmune thyroid disease in the family, obesity, drugs such as amiodarone or lithium, and geographical iodine deficiency [25]. The maternal thyroid gland produces the thyroid hormone, which crosses the placenta through a thyroid hormone transporter that is more selective for free thyroxine (fT4). Maternal fT4 is transformed into triiodothyronine (T3) and reverse triiodothyronine (rT3) in the presence of placental deiodinases. Then, before the embryonic thyroid grows, maternal T3 enters the fetal tissues [19]. Thyroid hormones hence significantly impact central nervous system development [19].

Mechanism of physiological changes of thyroid function in pregnancy and challenges

Throughout the first trimester, there is a rise in thyroxine-binding globulin (TBG) which is maintained at the same levels during the second trimester. This augmentation of TBG synthesis is caused by higher maternal estrogen levels, which also causes sialylation and, more critically, because of reduced clearance by the liver [1]. Because of an increase in maternal thyroid hormone production, there is an increase in concentration, resulting in a rise in total T3 and T4 levels, also causing a boost in the thyroid hormone production in the mother. Iodide is cleared from the kidneys when the maternal glomerular filtration rate increases. This clearance, together with enhanced thyroxine metabolism, leads to a drop in plasma iodide levels. Due to increased placental deiodinases, T4 metabolism is boosted in the second and third trimesters [2,26]. During pregnancy, the placenta is critical in reacting to and regulating the mother's thyroid hormones [27]. These alterations in levels of hormones induce changes in the size of the thyroid gland, which eventually reverts to its previous size after the baby is delivered. As a result, the measures used to assess hypothyroidism throughout pregnancy vary depending on the trimester and the remainder of the pregnancy [2,26]. Along with the thyroid gland, the placenta accumulates iodine. Iodine is required for the fetus to manufacture its thyroid hormones. Iodine is therefore taken up by iodine absorption from the mother [28]. Iodine inflow is assumed to be controlled by the sodium/iodine symporter (NIS), whereas outflow is controlled by pendrin [11,29].

Maternal hypothyroidism is linked to newborn growth, development, and intellect

The thyroid hormone promotes fetal development by promoting protein synthesis, RNA, DNA, and specific enzymes [30,31]. It also plays a vital role in tissue formation, maturation, and differentiation. The thyroid hormone is required for brain cell growth. Before the 20th week of pregnancy, brain development primarily relies on the mother's thyroid hormone. The brain proliferates throughout the first and second trimesters of pregnancy. Because fetal thyroid follicular epithelial cells are immature and cannot make thyroid hormone initially, this hormone is delivered to the fetus by transplacental administration [32,33]. Maternal thyroid hormone insufficiency in the latter stages of pregnancy may induce neurodevelopmental abnormalities, albeit the effects may be less severe than maternal thyroid shortage in the first trimester. Inadequacy is also found in levels of physical and intellectual development and response to environmental stimuli compared to children born to women with normal thyroid functioning throughout their pregnancy. Thyroid hormones are also responsible for forming long bones and teeth [30,32,33].

Outcomes of maternal hypothyroidism

Some symptoms of hypothyroidism, such as exhaustion, anxiety, constipation, muscular cramps, and weight gain, may be mimicked during pregnancy. Consequently, diagnosing hypothyroidism during pregnancy becomes more challenging [1,34,35]. Thyroid dysfunction may result in mental impairment and neurological disorders [36,37]. Pregnancy loss (miscarriage, intrauterine death, fetal loss), preterm labor (onset of labor before or after 37 weeks of gestation), preterm delivery (delivery before or after 37 weeks of gestation), gestational hypertension, preeclampsia, eclampsia, gestational diabetes, placental ligation performed by abruption (premature separation of a typically implanted placenta), and placenta previa may be some unfortunate expected outcomes. There has been evidence of poor response in terms of attention, language, reading motor, and visual-spatial skills in infants born to hypothyroid mothers [38]. One of the most prevalent outcomes in one of the cohort studies was neonatal jaundice. Reports of hypocalcemia, respiratory distress, down syndrome, cardiovascular abnormalities, and urogenital malformations have also been observed [39]. In addition, the offspring's decreased cognitive and motor functioning has also been seen [38,40].

Assessment

Hypothyroidism is thus a condition of an increased thyroid-stimulating hormone concentration with standard blood thyroxin (T4) levels (either total or free) or an elevated TSH concentration above 10mlU/L [7,37]. Multiple studies have been conducted across the globe to understand the effects of maternal hypothyroidism on fetal development; implications from certain such studies are put forth in Table 1.

**Table 1:** Implications from various studies (systemic review, meta-analysis, cohort study, prospective study, etc.) regarding effects of maternal hypothyroidism on fetal development.

Serial number	Name of author, month and or year (if any), citations	Type of Study	Objectives of the study	Implications
(1)	Lee et al., December 2022 [41]	Case report	To analyze dysfunction in the thyroid gland during pregnancy.	To avoid difficulties in fetal neurodevelopment, pregnant women with hypothyroidism must be treated with LT4.
(2)	Derakshan et al., June 2020 [23]	A systemic review and individual data meta-analysis	To assess the association of maternal thyroid functioning with birthweight.	Hypothyroidism in mothers during pregnancy is responsible for small gestational age and low birth weight in babies.
(3)	Parizad Nasirkandy et al., September 2017 [15]	Systemic review and meta-analysis	To assess the correlation of subclinical hypothyroidism during pregnancy and preterm birth.	Preterm birth was most commonly seen in mothers with subclinical hypothyroidism compared to euthyroid mothers.
(4)	Prezioso et al., 2018 [19]	Mini review	To analyze the dependency of neurodevelopment of fetus on thyroid hormones.	Maternal low thyroid functions are responsible for poor intellectual quotients and developmental retardation in infants. It is also linked to slower cognitive development in these infants.
(5)	Xu, Zhong, March 2022 [31]	Cohort study	To analyze and observe the association of hypothyroidism during pregnancy and its effect on fetal growth and development.	Hypothyroid mothers have a high risk of premature delivery, which may affect intellectual and psychomotor development in their infants.
(6)	Kankanamalage et al., May 2021 [11]	Randomized controlled trials	To understand the manner of development of gestational hypothyroidism.	Iodine deficiency during pregnancy and changes in estrogen in circulation is one of the common reasons that cause gestation hypothyroidism.
(7)	Kiran et al., September 2021 [39]	Cross-sectional retrospective study	To analyze and understand the anomalies caused in infants born to mothers with hypothyroidism.	The most common congenital anomalies included cardiovascular defects. Other abnormalities included low birth weight and neonatal jaundice.
(8)	Lucaccioni et al., September 2020 [38]	Prospective study	To assess the results seen in infants born to mothers having an abnormal functioning of the thyroid gland.	Maternal thyroid dysfunction may lead to fetal thyroid gland being dysfunctional with altered metabolism.
(9)	Ahmed, February 2015 [42]	A systemic review	To understand the effect of hypothyroidism in mothers on the brain development of their infants.	Lack of thyroid hormone in mothers during pregnancy is responsible for the retardation in brain development and decreased intelligence quotient in infants.

Treatment and management

During pregnancy, it is necessary to check the thyroid gland functions every 6 to 8 weeks [1]. Hypothyroidism in this condition should be treated to prevent obstetric complications and neurodevelopmental adversities in infants. Treatment includes administration of levothyroxine (LT4) sinceT3 cannot cross the placenta. Using T3 or a combination of T3 and T4 therapy may result in insufficient fetal thyroid hormone availability [41]. Pregnant women suffering from pre-existing hypothyroidism need to increase their pre-pregnancy dose of T4 to maintain the normal functioning of the thyroid gland [1]. Levothyroxine thyroid hormone (LT4) injection can be administered rarely and only in the setting of profound coma or impaired oral feeding after adjusting the dose [1,43,44]. Asymptomatic pregnant mothers in some countries are frequently screened for hypothyroidism [1]. Autoimmune hyperthyroidism is medically treated with methimazole or propylthiouracil, both anti-thyroid medications. Anti-thyroid medications may pass the placenta during pregnancy. If LT4 is appropriately dosed, most fetuses will get enough thyroid hormone levels throughout pregnancy. Maternal thyroid autoantibodies may cross the placenta but do not affect fetal or neonatal thyroid function. Neonates born to mothers with autoimmune hyperthyroidism may develop central hypothyroidism due to high fetal thyroid hormone concentrations inhibiting the hypothalamus-pituitary-thyroid axis [20,45,46]. Increased maternal iodine consumption is essential during pregnancy to provide adequate iodine levels for both mother and baby. With pregnancy, the recommended dietary allowance rises from 150 mcg to 250-300 mcg daily [11,43,44]. Thyrotropin, commonly known as TSH, is produced by the pituitary gland. It increases thyroid hormone synthesis and its release by the gland. Serum TSH levels rise when thyroid hormone concentrations are low and fall when concentrations are high. It is the standard screening test for thyroid dysfunction because it is a highly sensitive indicator of thyroid function [42,44].

## Conclusions

Hypothyroidism during pregnancy is a significant threat not only to the mother but also to her infant. There is a considerable risk of developmental anomalies and hindrances in the growth of infants in terms of intelligence and physical well-being. Since a pregnant mother can be diagnosed with hypothyroidism which can be present before she conceives or may develop it during her pregnancy, known as gestational hypothyroidism, it is necessary to identify the signs and symptoms as early as possible and get the required treatment. In cases of females who have had hypothyroidism since before must get treated before they plan to conceive. They must wait till the thyroid hormone levels get back to normal. After their levels reach the desired limit, women must wait for some time before they can conceive and get pregnant to prevent any harmful reactions that may hinder the baby's growth due to the presence of the drugs consumed for treatment. Mothers who develop gestational hypothyroidism must get treated as soon as they are diagnosed. Iodine is recommended for people living in areas with severe deficiency of iodine because of the inability and non-feasibility of salt iodization in such areas. Mothers must be educated and made aware of the importance of thyroid hormones and their functioning for their infant's proper growth and development. The knowledge about recognizing the early signs in themselves is of utmost importance. Regularly checking for normal levels of hormones in the blood should be encouraged, especially during the early phase of pregnancy. Monitoring and regulating the doses of prescribed medicines and maintaining normal functioning must be enabled.
